# Novel approach for reconstruction of the three-dimensional biliary system in decellularized liver scaffold using hepatocyte progenitors

**DOI:** 10.1371/journal.pone.0297285

**Published:** 2024-02-15

**Authors:** Kazuya Hirukawa, Hiroshi Yagi, Kohei Kuroda, Masafumi Watanabe, Kotaro Nishi, Shogo Nagata, Yuta Abe, Minoru Kitago, Shungo Adachi, Ryo Sudo, Yuko Kitagawa

**Affiliations:** 1 Department of Surgery, Keio University School of Medicine, Shinanomachi, Shinjuku, Japan; 2 Institute of Materials Science and Technology (E308), Technische Universität Wien, Vienna, Austria; 3 Department of System Design Engineering, Keio University, Kohoku-ku, Yokohama, Japan; 4 Molecular Profiling Research Center for Drug Discovery, National Institute of Advanced Industrial Science and Technology, Tokyo, Japan; University of Navarra School of Medicine and Center for Applied Medical Research (CIMA), SPAIN

## Abstract

Reconstruction of the biliary system is indispensable for the regeneration of transplantable liver grafts. Here, we report the establishment of the first continuous three-dimensional biliary system scaffold for bile acid excretion using a novel method. We confirmed the preservation of the liver-derived extracellular matrix distribution in the scaffold. In addition, hepatocyte progenitors decellularized via the bile duct by slow-speed perfusion differentiated into hepatocyte- and cholangiocyte-like cells, mimicking hepatic cords and bile ducts, respectively. Furthermore, qRT-PCR demonstrated increased *ALB*, *BSEP*, and *AQP8* expression, revealing bile canaliculi- and bile duct-specific genetic patterns. Therefore, we concluded that locally preserved extracellular matrices in the scaffold stimulated hepatic progenitors and provided efficient differentiation, as well as regeneration of a three-dimensional continuous biliary system from hepatic cords through bile ducts. These findings suggest that organ-derived scaffolds can be utilized for the efficient reconstruction of functional biliary systems.

## Introduction

Liver transplantation is the only reliable treatment for patients with end-stage liver disease; however, the shortage of liver donors remains a challenge. Regenerative medicine is a major approach to solving this issue, and alternative long-term liver grafts should possess the major functions of the native liver, including the production and secretion of bile, protein synthesis, metabolic function, and detoxification. Cholestatic diseases, such as biliary atresia, primary sclerosing cholangitis, and progressive familial intrahepatic cholestasis (PFIC), have a poor prognosis due to dysfunction of bile secretion; consequently, the bile secretion function cannot be compensated or treated with medication [1–3]. Thus, the reconstruction of the biliary system is indispensable for the regeneration of transplantable liver grafts.

Few studies have reported reconstruction of the biliary system with functional heterogeneity methods, primarily due to the complexity of the biliary system [4, 5]. The bile canaliculi are the smallest bile ducts formed by the apical membrane of two adjacent hepatocytes. These hepatocytes display a membrane polarity of specific transporter proteins, such as bile salt export pump (BSEP), the abnormality of which causes PFIC type II and cystic fibrosis transmembrane conductance regulator (CFTR). Bile canaliculi are organized and integrated into large bile ducts covered with cholangiocytes expressing the secretin receptor (SCTR), carbonic anhydrase 2 (CAR2), and aquaporin 8 (AQP8). Three-dimensional (3D) cell culture techniques, such as sandwich or chip cultures, only mimic the partial ductal structures of the biliary system and lack the heterogeneity in size and functions described above [6–8]. Koike et al. developed a hepato-biliary-pancreatic integrated morphogenesis model using foregut–midgut boundary organoids [9]. This approach enables biological engineering techniques to reconstruct higher-order developmental events, including long-range tissue patterning and morphodynamics, tissue interactions, and organism-level organizations and functions. However, the reconstructed tissue is limited to the laboratory scale and is not sufficiently large for clinical use as an organ graft.

Decellularized liver scaffolds preserve the native organ size and organ-specific structures, including ductal structures, enabling scaffolds to be anastomosed with recipient vessels or bile ducts. A previous study demonstrated the utility of decellularized liver scaffolds as transplantable regenerative organs in long-term experiments using large animal models of chronic liver dysfunction [10]. In addition, the liver-derived extracellular matrix (ECM) is preserved in the scaffold, contributes to adhesion, morphology, and differentiation of perfused cells, and regulates the activation of progenitor or mature liver cells as a hepatocyte progenitor niche [11–14].

Primary cholangiocytes are cumbersome to expand *in vitro* and difficult to use in clinical trials due to insufficient availability from recipients, a major roadblock in the recellularization of the scaffold biliary duct. One solution is to use induced pluripotent stem (iPS) cells. A recently published study showed successful transplantation of decellularized liver grafts recellularized by human iPS cell-derived hepatocytes in a rat model [15]. Human iPS cells are a promising component for recellularization in terms of pluripotency and proliferation capacity. However, concerns regarding the difficulty of culture, cost, and uncertainty of differentiation remain. Hepatocyte progenitors were first described by Farber et al., who showed the proliferation of oval cells and ductular reactions [16]. Further investigation revealed the bipotentiality of hepatocyte progenitors under defined conditions [16–18]. Recently, liver progenitors have shown high proliferation potential and the ability to differentiate into hepatocytes and cholangiocytes [19, 20]. Since the recellularization of biliary ductular construction is achieved by reepithelization of the large bile duct with cholangiocytes and integration with bile canaliculi organized by hepatocytes, generating cholangiocytes and hepatocytes is essential for bile duct reconstruction. The bipotentiality of hepatocyte progenitors may prove useful for reconstructing the continuous biliary system, including bile canaliculi and large bile ducts in the scaffold.

Here, we report a novel method for the 3D reconstruction of the continuous biliary system from bile canaliculi to large bile ducts by recellularizing progenitor hepatocytes in decellularized liver scaffolds. The morphology and gene expression of progenitor hepatocytes in recellularized livers suggest that progenitor hepatocytes are a promising cell resource for reconstructing the biliary system.

## Materials and methods

### Animals

All experimental procedures and protocols were approved by the Animal Ethics Committee of the Keio University School of Medicine (approval number: 09215). The animals were treated according to the guidelines of the Ministry of Education, Culture, Sports, Science and Technology, Japan.

Female Lewis rats (250–300 g; Sankyo Labo Service Corp., Inc., Tokyo, Japan.) were used for harvesting liver to prepare decellularized scaffolds and to isolate hepatocytes for recellularization. The animals were anesthetized with 2–4% isoflurane mask inhalation to maintain anesthesia during the procedure. The vena cava was cut, which caused exsanguination of the animal under deep surgical anesthesia.

### Preparation of liver-derived ECM scaffolds

**Fig 1:**
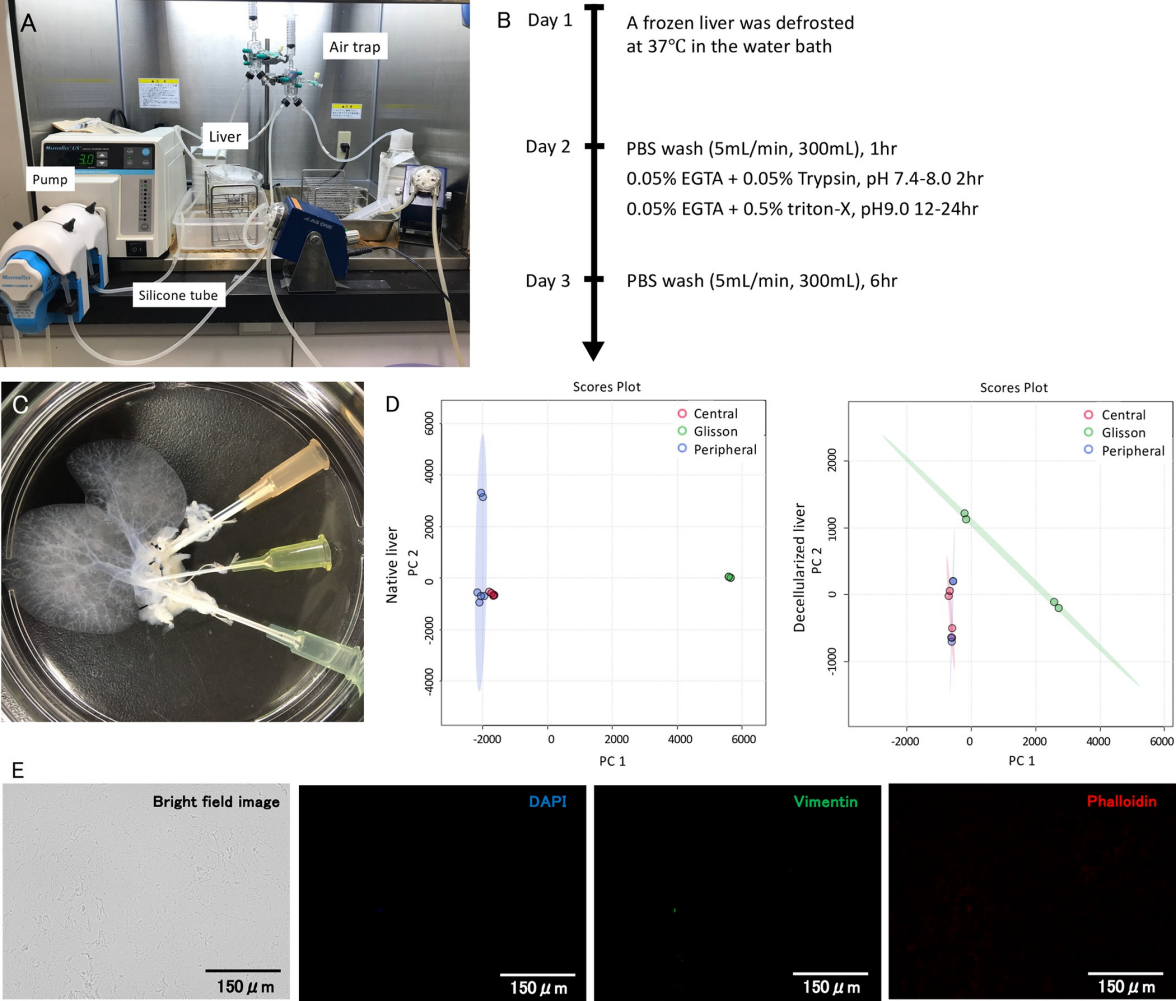
Decellularization protocol and protein assay. (A) Photograph illustrating the organ perfusion chamber used to decellularized rat liver. (B) Decellularization protocol of rat liver. (C) Decellularized rat liver. Small caudate lobes, left lobe and the right small lobe were removed to reduce the volume and catheters were inserted into each duct. (D) Protein assay of native liver and decellularized liver. Scatter plot shows principal component analysis of protein components in native liver and decellularized liver scaffold.

Rat livers were harvested using the method described by Yagi et al [21]. The liver-derived ECM scaffolds were created by continuous detergent perfusion of the entire dissected rat liver. Briefly, the frozen livers were thawed at 4°C and subsequently washed with phosphate-buffered saline (PBS; FUJIFILM Wako Pure Chemical Corp. Osaka, Japan) to remove blood through perfusion via the portal vein. Decellularization was achieved by perfusing the liver with 0.05% ethylene glycol tetraacetic acid (EGTA; Tokyo Chemical Industry Co. Ltd. Tokyo, Japan) and 0.05% trypsin (FUJIFILM Wako Pure Chemical Corp. Osaka, Japan) in deionized water, followed by EGTA (0.05%) and 0.5% Triton X (Thermo Fisher Scientific K.K., Tokyo, Japan). The liver scaffold was washed extensively with sterile PBS and preserved in PBS supplemented with antibiotics at 4°C for up to 7 days (Fig 1A and 1B). An air trap was set between the liver and pump to remove air bubbles. At the time of harvesting, the 22-, 24-, and 16-G catheters were inserted into the portal vein, bile duct, and inferior vena cava (IVC), respectively, for recellularization and continuous perfusion of the scaffold (Fig 1C). The catheters were cut to adequate lengths in advance to prevent damage to the ducts and cannulation of peripheral branches. Caudate, left, and right small lobes were resected to reduce the volume of the scaffold needed to be repopulated. The Glissonean pedicles and hepatic veins at the dissected plane were ligated using 4–0 silk suture to prevent leakage of perfusates. A homogeneously decellularized liver scaffold was acquired using a catheter inserted into the bile duct.

The two principal components explaining the highest variance were plotted on X and Y axis. Blue and red dots represent the samples from central and peripheral parenchymal area of the samples, and green dots represents the samples from Glissonean pedicles, indicating preservation of similarity of protein distributions before and after decellularization. (E) The bright field images and immunofluorescent images following staining for nuclei, intermediate filament, and actin showing complete absence of cellular components within the decellularized scaffold.

### Protein assay examination and histological analysis of decellularized scaffold

Native rat liver and decellularized rat liver scaffolds were cut into 10-mg pieces in the central and peripheral hepatic parenchyma and Glisson of the hilar area. Each piece was digested and peptided in a solution of 0.001% trypsin, 10 μM Tris-HCl, 0.005% n-octyl glucopyranoside, and 0.7 M guanidine hydrochloride (pH 8.8) for 12 h. Thereafter, they were reduced with 5 mM tris(2-carboxyethyl) phosphine for 30 min at 65°C. Following alkylation with 10 mM iodoacetamide for 30 min at 25°C, samples were washed on a C18 monospin column (GL Science K.K., Tokyo, Japan). Purified peptides (500 ng) were separated using a C18 separation column (Nikkyo Technos Co. Ltd., Tokyo, Japan) and analyzed using an Easy-nLC 1200 coupled with a Q-Exactive HF-X instrument (Thermo Fisher Scientific K.K., Tokyo, Japan) in data-dependent acquisition mode, where the top 25 recorded mass spectrometry spectra between 380 and 1500 m/z were selected. Survey scans were acquired at a resolution of 60,000 at m/z 200, and the tandem mass spectrometry (MS/MS) resolution was set to 15000 at m/z 200. All MS/MS spectra were searched against the protein sequences of the Sus scrofa (NCBI: txid9823) protein database using Proteome Discoverer 2.2 (Thermo Fisher Scientific K.K., Tokyo, Japan) with the SEQUEST search engine. The data matrix was statistically arranged using Metabo-Analyst (https://metaboanalyst.ca/), an online platform for reading metabolomic data using default parameters.

Immunofluorescence analysis was performed to detect the presence of nuclei, actin, and intermediate filaments in the residual cells of the decellularized scaffold.

### Isolation of rat hepatocytes and cholangiocytes and establishment of hepatocyte progenitors

**Fig 2:**
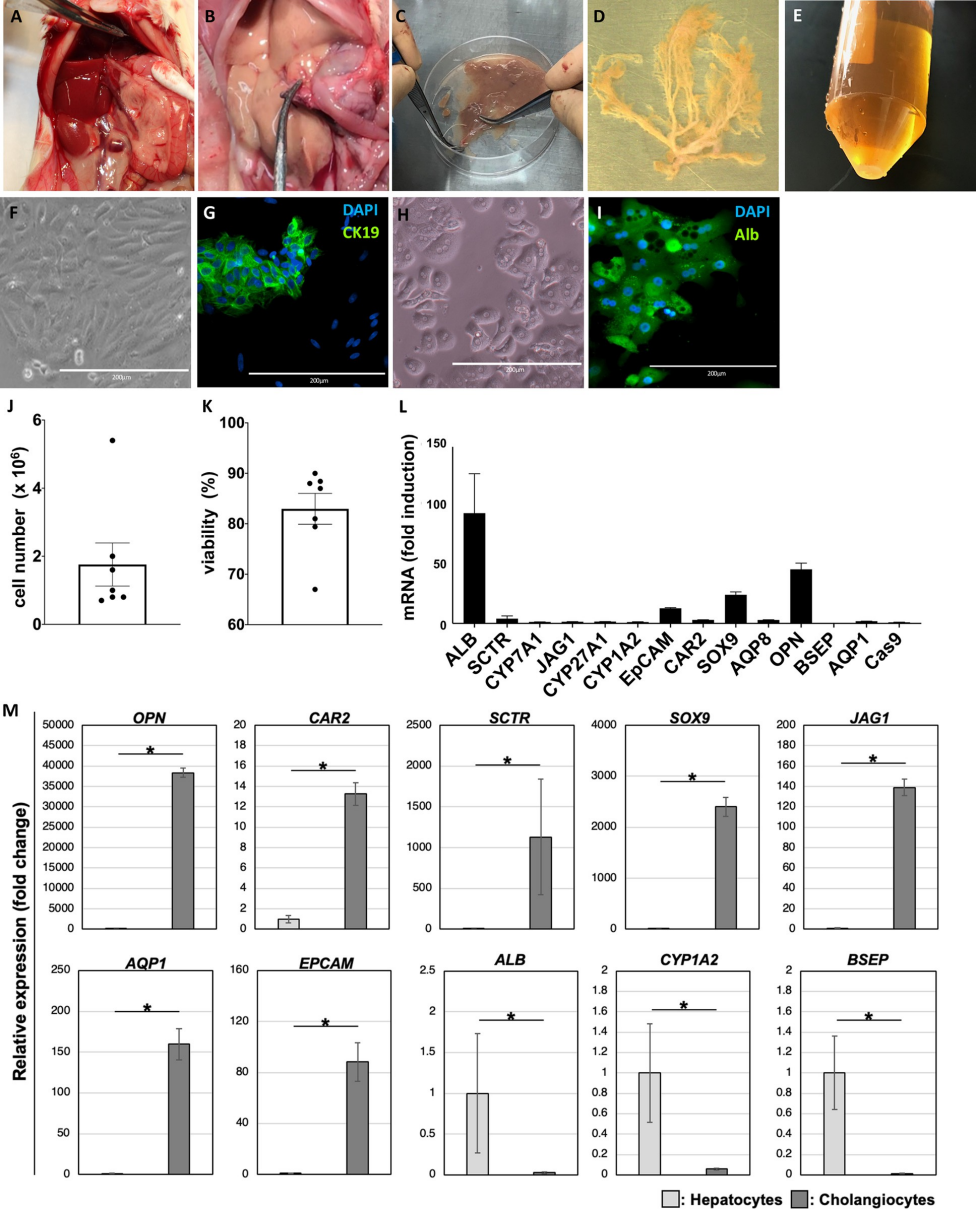
Harvest of cholangiocytes from rat liver. (A) Mobilization of liver and exposure of hepatoduodenal ligament before insertion of catheters. (B) Ligation and dissection of common bile duct and after perfusion of perfusate including collagenase. (C) Removal of liver parenchymal tissue. (D) Biliary tree. (E) Primary cholangiocytes were obtained as cell pellet following centrifugation. (F, G) Microscopic image and immunostaining using anti-CK19 antibody for hepatocytes. (H, I) Microscopic image and immunostaining using anti-ALB antibody for hepatocytes. (J) Quantification of the cell number of cholangiocytes. (K) Quantification of the cell number of cholangiocytes by TUNEL staining (L) Relative gene expression of primary cholangiocytes normalized by *CAS9*. Bars represent the mean ± SD, n = 3. (M) Comparison of gene expression in isolated hepatocytes and bile duct epithelial cells. Bars represent the mean ± SD, n = 3.

Cholangiocytes and hepatocytes were isolated as follows. First, a rat liver was exposed and mobilized (Fig 2A), and the portal vein, proper hepatic artery, and common bile duct were ligated using a 4–0 silk thread. A 20-G catheter was inserted into the portal vein, and 150 mL of the primary perfusate (ethylene glycol tetraacetic acid (EGTA) solution (distilled water (DW) 441 mL, EGTA 0.095 g, 10× Hanks’ Balanced Salt Solution (HBSS; FUJIFILM Wako Pure Chemical Corp. Osaka, Japan) 50 mL, 10 mg/mL Insulin (Sigma-Aldrich Japan, Tokyo, Japan) 25 μL, 1 M NaHCO_3_ (FUJIFILM Wako Pure Chemical Corp. Osaka, Japan) 3.5 mL, and 100× penicillin-streptomycin (Nacalai Tesque, INC., Kyoto, Japan) 5 mL, pH 7.5) was perfused at the speed of 30 mL/min immediately after IVC was dissected (Fig 2B). The rat liver was resected and underwent perfusion with the second perfusate (Hanks’ solution (431 mL DW, 50 mL of 10× HBSS, 100 mM CaCl_2_.2H_2_O (FUJIFILM Wako Pure Chemical Corp. Osaka, Japan) 5 mL, 100 mM MgSO_4_.7H_2_O (FUJIFILM Wako Pure Chemical Corp. Osaka, Japan) 5 mL, 25 μL of 10 mg/mL insulin, 3.5 mL of 1 M NaHCO_3_, and 5 mL of 100× penicillin-streptomycin, pH 7.5) 200 mL, and 50 mg collagenase (Sigma-Aldrich Japan, Tokyo, Japan) at a speed of 15 mL/min. The liver capsule was then removed from the plate filled with 500 mL of L-15 solution (L-15 (Thermo Fisher Scientific K.K., Tokyo, Japan), 30 μL of 10 mg/mL insulin, 20 μL dexamethasone (Sigma-Aldrich Japan, Tokyo, Japan), 2.38 g of 2-[4-(2-Hydroxyethyl)-1-piperazinyl]ethanesulfonic acid (HEPES; FUJIFILM Wako Pure Chemical Corp. Osaka, Japan), 15 mg of L-proline (FUJIFILM Wako Pure Chemical Corp. Osaka, Japan), and 0.55 g of galactose (FUJIFILM Wako Pure Chemical Corp. Osaka, Japan), and the liver was then swinged. The liver parenchyma crumbled and sank (Fig 2C). The remnant tissue was rinsed with L-15 solution using a 250-μm filter. The rinse was collected as clusters of hepatocytes, and a biliary tree was obtained (Fig 2D). Clusters of hepatocytes were filtered using a 100-μm cell strainer, and the filtrate was centrifuged for 3 min at 50 *×g*. After the supernatant was removed, 25 mL L-15 solution (10× HBSS 2.5 mL and Percoll (Sigma-Aldrich Japan, Tokyo, Japan) 22.5 mL) was added and centrifuged for 10 min at 100 ×*g* at 4°C. The supernatant was removed and rinsed with L-15 solution. Hepatocytes were isolated by centrifugation for 3 min at 50 g. After the biliary tree was shaken for 10 min in collagenase solution (L-15 25 mL, collagenase 15 mg), it was minced using scissors for 20 min in L-15 solution. Collagenase solution (L-15 25 mL, collagenase 20 mg, hyaluronidase (Sigma-Aldrich Japan, Tokyo, Japan) 21 mg, and trypsin inhibitor (FUJIFILM Wako Pure Chemical Corp. Osaka, Japan) 2.5 mg), and was stirred in a beaker for 50 min. The tissue was filtered using 100-, 40-, and 20-μm cell strainers and centrifuged twice for 10 min at 850 ×*g*. The sediment containing cholangiocytes was collected (Fig 2E). The suspension of the recovered cholangiocytes was mixed with 0.3% trypan blue stain (1:1), and the total number of cells and dead cells were counted using a blood cell calculator to calculate viability.

Hepatocyte progenitors were generated using the method described by Katsuda et al [19]. Briefly, 10 μM Y-27632 (FUJIFILM Wako Pure Chemical Corp. Osaka, Japan), 0.5 μM A-83-01 (FUJIFILM Wako Pure Chemical Corp. Osaka, Japan), and 3 μM CHIR99021 (FUJIFILM Wako Pure Chemical Corp. Osaka, Japan) (YAC) were added to isolated rat hepatocytes using the method described above. The treated cells were seeded on plates coated with collagen type I (KOKEN CO., LTD, Tokyo, Japan) at 1 × 10^4^ cells/cm^2^. The medium was changed 1 day after seeding and then every other day before recellularization.

### Recellularization and culture protocol

Cholangiocytes were diluted in 500 μL Dulbecco’s modified Eagle’s medium (DMEM) (DMEM (Thermo Fisher Scientific K.K., Tokyo, Japan) 500 mL, fetal bovine serum (FBS; JR Scientific Inc., woodland, CA, USA) 50 mL, dexamethasone 22 μL, gentamycin (Thermo Fisher Scientific K.K., Tokyo, Japan) 550 μL, insulin 550 μL, 1 M nicotinamide (Sigma-Aldrich Japan, Tokyo, Japan) dissolved in PBS 5.5 mL, 100 mM ascorbic acid (Sigma-Aldrich Japan, Tokyo, Japan) dissolved in PBS 5.5 mL, 5 mg/mL transferrin (Thermo Fisher Scientific K.K., Tokyo, Japan) 550 μL, epidermal growth factor (EGF; Corning Incorporated, New York, America) 55 μL, and hepatocyte growth factor (HGF; R&D Systems, Inc., Minneapolis, America 110 μL) and disseminated continuously for 5 min in the scaffold via a catheter inserted into the bile duct immediately after harvesting. After dissemination of cholangiocytes into the scaffold, the scaffold was continuously perfused via the bile duct at a the rate of 1) 300 μL/min and 2) 10 μL/min with a Micro Tube pump System (ICOMES LAB Co., Ltd. Tokyo, Japan). Hepatocyte progenitors were diluted with 1000 μL EDM and disseminated continuously for 10 min in the scaffold via a catheter inserted into the bile duct immediately after being stripped from the culture dishes. After dissemination of hepatocyte progenitors into the scaffold, the scaffold was continuously perfused via the bile duct at the rate of 300 μL/min for the first 3 days followed by 10 μL/min for 11 consecutive days with the Micro Tube pump System.

### Histology and immunofluorescence analysis of recellularized scaffold

After continuous perfusion culture, a histological analysis was performed to evaluate the degree of cell infiltration into the sutured scaffolds. Tissue samples were fixed with 4% paraformaldehyde in PBS. The fixed samples were paraffin-embedded and sectioned at 0.5–1 μm thickness. Sections were stained with hematoxylin and eosin (HE) following standard protocol.

Isolated cholangiocytes were fixed in 5% dimethyl sulfoxide in methanol and subsequently blocked with 5% FBS in PBS. Fixed samples were stained overnight at 4°C with anti-rat cytokeratin-19 (CK19) (ab84632, Abcam Inc., Cambridge, UK) as the primary antibody. The primary antibody used for the isolated hepatocytes was anti-rat albumin (ALB) (ab8940; Abcam Inc., Cambridge, UK). For the established hepatocyte progenitors, the primary antibodies were anti-rat ALB (ab8940, Abcam Inc., Cambridge, UK) and CK19 (ab84632, Abcam Inc., Cambridge, UK). For the recellularized scaffold with cholangiocytes, the primary antibody used was anti-rat CK19 (ab84632, Abcam Inc., Cambridge, UK). The decellularized scaffold with hepatocyte progenitors was analyzed using the following primary antibodies: anti-rat ALB (ab8940, Abcam Inc., Cambridge, UK), hepatocyte nuclear factor 4 alpha (HNF4α) (ab201460, Abcam Inc., Cambridge, UK), alpha fetoprotein (AFP) (ab46799, Abcam Inc., Cambridge, UK), CK19 (ab84632, Abcam Inc., Cambridge, UK), CFTR (ab2784, Abcam Inc., Cambridge, UK), AQP1 (20333-1-AP, Proteintech, Tokyo, Japan) and ZO-1 (33–9100, Thermo Fisher Scientific Inc., Waltham, MA, USA).

The secondary antibodies for HNF4α, AFP, and CK19 were goat anti-rabbit IgG Alexa Fluor 488 (ab96899; Abcam Inc., Cambridge, UK). The secondary antibody for ALB was goat anti-sheep IgG Alexa Fluor 488 (A11015; Invitrogen Inc., Carlsbad, CA, USA). The secondary antibody for CFTR was goat anti-mouse IgM Alexa Fluor 488 (A21402; Invitrogen Inc., Carlsbad, CA, USA). The secondary antibody for AQP1 was donkey anti-rabbit IgG Alexa Fluor 488 (ab150073; Abcam Inc., Cambridge, UK). The secondary antibody for ZO-1 was goat anti-mouse IgG Alexa Fluor 488 (ab150113; Abcam Inc., Cambridge, UK). Counter staining was performed with a 4′,6-diamidino- 2-phenylindole (DAPI)-containing mounting medium (P36971, Invitrogen Inc., Carlsbad, CA, USA).

### Evaluation of cholangiocyte alignment in perfusion culture

Recellularized liver samples were fixed in 4% paraformaldehyde at 4°C overnight and then permeabilized with 0.1% Triton X-100 overnight. After rinsing with PBS, the samples were treated with Block Ace (Sumitomo Pharma Co., Ltd., Tokyo, Japan) overnight to inhibit nonspecific staining. Samples were then incubated with Alexa Fluor 594-conjugated phalloidin (1:100 dilution; Thermo Fisher Scientific Inc., Waltham, MA, USA) and Hoechst 33342 (1:500 dilution; Thermo Fisher Scientific Inc., Waltham, MA, USA) to stain actin filaments, intermediate filament and nuclei, respectively. Finally, the samples were rinsed with PBS. To obtain fluorescence images, the samples were placed in a glass-bottomed dish (No. 1; AGC Techno Glass Co., Ltd., Shizuoka, Japan). Fluorescence images were obtained using a confocal laser scanning microscope (LSM700; Carl Zeiss AG, Oberkochen, Germany) with a 20× objective lens. The 3D images were generated with z-stack fluorescent images using ImageJ (National Institutes of Health, Bethesda, MD, USA) or Imaris 7.7.0 software (Bitplane, Belfast, UK).

### Gene expression analysis

The samples were immediately submerged in RNAlater Stabilization Solution (Thermo Fisher Scientific K.K., Tokyo, Japan) for gene expression analysis and stored at -20°C until total RNA extraction. The tissue was homogenized, total RNA was isolated using a commercially available column-based purification kit (RNeasy Mini Kit, QIAGEN K.K. Tokyo, Japan) according to the manufacturer’s instructions, and the RNA content was measured using a NanoDrop spectrophotometer (Thermo Fisher Scientific K.K., Tokyo, Japan) spectrophotometer (Thermo Fisher Scientific K.K.). Complementary DNA (cDNA) was synthesized from 1 μg total RNA per sample using PrimeScript RT Master Mix (Takara Bio Inc., Shiga, Japan) according to the manufacturer’s instructions. Quantitative RT-PCR (qRT-PCR) was performed using Power SYBRTM Green PCR Master Mix (Thermo Fisher Scientific K.K., Tokyo, Japan) and the ViiA7 Real-Time PCR System (Thermo Fisher Scientific K.K., Tokyo, Japan) according to the manufacturer’s instructions. GAPDH was used as an internal control for the comparative CT method. Hierarchically clustered heatmap was created using GraphBio (http://www.graphbio1.com/en/#) based on Z-scores of the normalized ΔCT values.

### Statistical analyses

Data were means ± SD. For qRT-PCR, the relative gene expression was calculated using the comparative ΔΔCt for several independent experiments. For comparing two mean values, a two-sided Student’s *t-*test was performed to calculate statistical significance, which was set at P < 0.05. The analyses were performed using SPSS version 26.0 (IBM Co., Armonk, NY, USA).

## Results

### Protein array of the liver scaffold

A protein assay of whole rat decellularized liver was performed by Takeishi et al. [22], however, they merely suggested that the scaffold retains proteins such as growth factors and mediators, but did not elaborate on whether the proteins preserved individual localization in each area of liver. In this study, characteristics of proteins localization in each part of the native liver and scaffold (central and peripheral parts of the liver parenchyma, and Glissonean pedicle (perivascular part)) were assessed by performing principal component analysis (Fig 1D). The protein components landscape of Glissonean pedicle (green dot) was distinct from those from central (blue dot) and peripheral (red dot) liver parenchyma. The same distinction was observed in the analysis of scaffolds, indicating that the localization of ECM proteins in the liver were preserved after decellularization (Fig 1D). Immunostaining results for nuclei, intermediate filament, and actin were all negative, showing absence of any cellular components (Fig 1E).

### Simultaneous isolation protocol of primary cholangiocytes and hepatocytes

To determine the appropriate method of perfusion and circulation in ductal structures originating from bile ducts, isolated cholangiocytes were used to recellularize the liver scaffold via the bile duct. As cholangiocytes cannot be maintained *in vitro*, we isolated cholangiocytes immediately before their dissemination into the scaffold. In the process of obtaining the bile duct tree (Fig 2A–2E), the liver parenchymal cells that were removed from the bile duct tree (Fig 2C) were isolated and used for the subsequent establishment of the hepatocyte progenitor. Immunohistological imaging showed that the isolated cholangiocytes were spindle shaped and expressed CK19 (Fig 2F and 2G). The average cell number and viability of isolated cholangiocytes were 1.757 × 10^6^ ± 1.677 and 82.97 ± 8.084%, respectively (n = 7). Primary hepatocytes, which were collected simultaneously, showed cuboidal morphology, round nuclei, and expressed ALB (Fig 2H and 2I). In the RT-qPCR analyses of cholangiocytes (Fig 2L), the expression of cholangiocyte-specific markers such as SCTR, EpCAM, OPN, and SOX9 was high (4.074-, 12.867-, 45.974-, and 24.312-fold, respectively). On the contrary, the expression of bile canaliculi- and large bile duct-specific markers such as BSEP and AQP8 was low (0.083- and 2.950-fold, respectively). Comparison of gene expression in isolated hepatocytes and cholangiocytes showed significantly higher expression of genes specifically expressed in each cell lineage (Fig 2M).

### Slow speed perfusion culture of primary cholangiocytes

**Fig 3:**
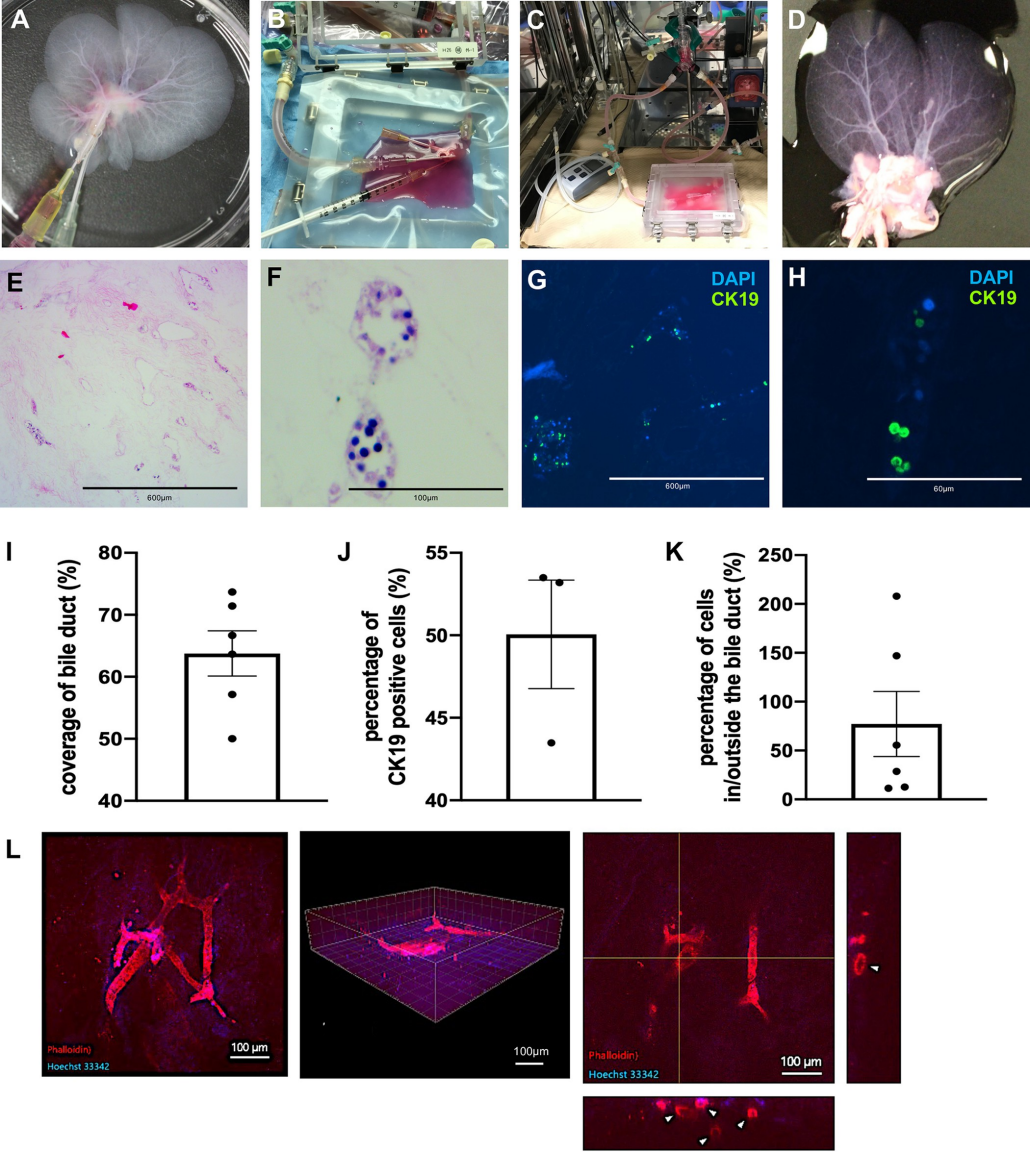
Recellularization of primary cholangiocytes via the bile duct with slow-speed perfusion culture. (A) Before connecting the circulation machine, the ducts were stained using perfusion fluid (pink) to ensure the catheter was correctly inserted into the biliary ductal structure. (B) Cholangiocytes were slowly injected into the ductal structure via the catheter inserted into the extrahepatic bile duct. (C) The scaffold is packed in the chamber after recellularization and continuously perfused. (D) Macroscopic image of scaffold after perfusion and culture. (E, F) HE staining of the scaffold recellularised with cholangiocytes. The recellularised cholangiocytes repopulated in the bile ducts of various sizes and cubic columnar cholangiocytes aligned on the ductal walls. (G, H) Immunohistological images of the scaffold stained with anti-CK19 antibody. (I, J, K) The ratio of the number of bile ducts covered with cholangiocytes/the number of bile ducts in the field, percentage of CK 19 positive cells/the whole number of cells in the field, and percentage of cells in/outside the ductal structures. Bars represent the mean ± SD. (L) Immunofluorescence images of constructed ductal networks in recellularised liver scaffolds with cholangiocytes using a confocal laser scanning microscope. The cholangiocytes attached to the inner surface of the bile duct.

After decellularization, DMEM was perfused through the catheter cannulated into the bile duct to visualize it (Fig 3A) and ensure that the catheter was correctly inserted into the biliary tract. Next, the scaffolds were placed in a perfusion chamber. To avoid filtration or damage to the ductal structure of the scaffold by dissemination of cholangiocytes, we perfused 500 μL cholangiocyte suspension (≤ 1.0 × 10^4^ cells/μL). After distributing the primary cholangiocytes into the scaffold (Fig 3B), the chamber was sealed, and continuous perfusion culture was initiated (Fig 3C). After 3-day culture, the scaffold was retrieved from the chamber (Fig 3D). The speed of circulation was initially set at 300 μL/min, resulting in cholangiocyte distribution outside the bile ducts. We then slowed down the perfusion speed to 10 μL/min and found CK19-positive cholangiocytes aligning and covering the bile ducts (Fig 3E–3H). The quantitative analysis showed that CK19-positive cholangiocytes broadly covered bile ducts (63.76 ± 8.932% of ductal structures, Fig 3I). The ratios of CK19-positive cells to total cells and distribution of cells inside and outside the duct structures at six randomly selected sites were 50.05 ± 5.696 and 77.17 ± 81.67%, respectively (Fig 3J and 3K). These results indicated that cholangiocytes infiltrated the parenchymal area and covered the bile ducts. Immunofluorescence staining results obtained using a confocal laser scanning microscope are shown in Fig 3L. The constructed continuous dendritic networks were observed in the liver scaffolds recellularized with cholangiocytes. The lumens of the ductal structures were not filled with injected cells, and only the inner lumen surface was covered with cholangiocytes (white arrowhead). The cross-sections of the confocal image showed that the thinnest diameter of the ductal structures covered with cholangiocytes was 10 μm, which corresponds to the size of the peripheral bile duct, and that the ductal structures covered with cholangiocytes spread three-dimensionally. Importantly, we succeeded in reconstructing not only large intrahepatic bile ducts but also peripheral ductal structures using our perfusion method.

### Recellularization and maturation of hepatocyte progenitors in the liver scaffold

**Fig 4:**
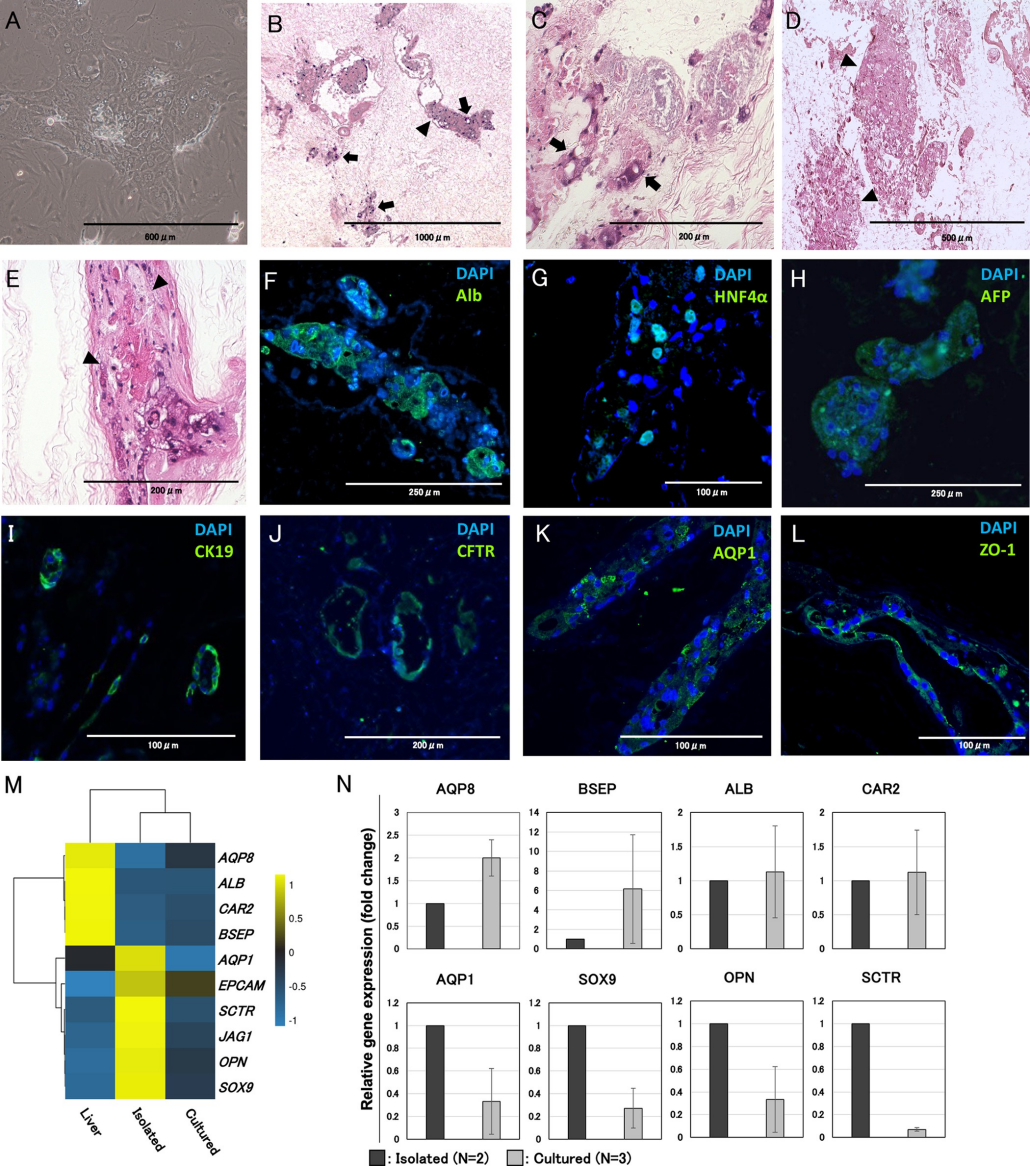
Histological and genetic analysis findings showed differentiation of chemically-induced liver progenitors into hepatocytes and cholangiocytes. (A) Microscopic image of hepatocyte progenitors. (B–E) HE staining of scaffold recellularization and perfusion culture for 14 days with hepatocyte progenitors. Infiltration of hepatocyte-like cells (black arrowhead) into the interstitial field and coverage of bile ducts with cholangiocyte-like cells (black arrow) were observed. (F–L) Immunohistological images of the scaffold stained using anti-ALB, anti-HNF4α, anti-CK19, anti-AFP, anti-CFTR, anti-AQP1 and anti-ZO-1 antibodies. (M) Heatmap showing expression profile of hepatocyte- and cholangiocyte-specific genes in liver tissues (Liver), isolated hepatocyte progenitors (Isolated), and hepatocyte progenitors cultured in the scaffold (Cultured). (N) Relative gene expressions of isolated hepatocyte progenitors (n = 2) and hepatocyte progenitors cultured in the scaffold (n = 3). Bars represent the mean ± SD.

Before recellularization of hepatocyte progenitors to the liver scaffold, we examined their morphological characteristics. Hepatocyte progenitors showed a high nucleus/cytoplasm ratio and included binucleated cells, which was consistent with the results of the original study [19] (Fig 4A). Cells were continuously passaged and proliferated in YAC(+) culture medium, and the morphology of hepatocyte progenitors was preserved through repeated subculture. Hepatocyte progenitors were obtained by trypsin treatment and suspended in 1000 μL perfusion medium (YAC(-)). Similar to the recellularization of cholangiocytes, hepatocyte progenitors were distributed into the liver scaffold via bile ducts and then cultured with continuous perfusion (at 300 μL/min for the first 3 days and 10 μL/min for the next 11 days.) After continuous perfusion culture, the scaffolds were retrieved and analyzed using hematoxylin and eosin (HE) and immunofluorescence staining. HE staining showed that bile ducts were covered with simple columnar cholangiocyte-like cells (Fig 4B and 4C, black arrow), while hepatocyte-like cells characterized by cuboidal morphology and round nuclei infiltrated and aligned in a manner similar to the original hepatic cords in parenchymal areas (Fig 4B, 4D, 4E, black arrowhead). Immunofluorescence images revealed the expression of hepatocyte-specific markers such as ALB, HNF4α, and AFP in hepatocyte progenitors repopulating the parenchymal areas (Fig 4F–4H). In contrast, hepatocyte progenitors in the ductal structures expressed cholangiocyte-specific markers, such as CK19, CFTR, AQP1, and ZO-1 (Fig 4I–4L). These results indicated that hepatocyte progenitors in parenchymal areas differentiated into hepatocytes and those in ductal structures differentiated into cholangiocytes through the interaction between hepatocyte progenitors and ECM-derived factors. Heatmap of hierarchical clustering is shown in Fig 4M. Compared to the gene expression profile of the native liver tissue, the expression of genes specific to the cholangiocyte lineage was relatively high in cultured progenitors. A comparison between isolated progenitors and cultured progenitors revealed relative suppression of the cholangiocyte lineage specific genes, while the expression of the hepatocyte lineage specific genes was increased. This suggested that the progenitors differentiated during the culture process. Using RT-qPCR analyses (Fig 4N), we analyzed the expression of markers specific to mature hepatocytes (*ALB)*s, bile canaliculi (*AQP8*, *BSEP* and *CAR2)*, and bile duct (*AQP1*, *SOX9*, *OPN* and *SCTR*) in cultured progenitors (n = 3) and isolated progenitors (n = 2). While the relative expression of *AQP1*, *SOX9*, *OPN* and *SCTR* was relatively low in cultured progenitors and *ALB* expression was comparable, the expression of *BSEP* and *AQP8* was relatively high in cultured progenitors compared to isolated hepatocyte progenitors. These results indicated that the scaffold with preserved ECM proteins expressed in the ductal structures and parenchymal areas enhanced the expression of functional genes specific to bile canaliculi and the functional construction of lined hepatocytes connected to bile ducts, without external addition of stimulating factors.

## Discussion

Here, we present a two-step perfusion culture of hepatocyte progenitors using a decellularized liver scaffold that successfully distributed progenitors in the parenchymal area and biliary ductal structure of the scaffold. Hepatocyte progenitors in the scaffold differentiated into hepatocytes and cholangiocytes without additional external stimulating factors and morphologically mimicked hepatic codes and a continuous structure from hepatic codes to bile ducts. In addition, the results of qRT-PCR supported the reconstruction of several sizes of bile ducts from bile canaliculi to intralobular bile ducts. Although earlier studies have assessed the optimal recellularization in intralobular bile ducts in a decellularized liver scaffold [22–24], none of them analyzed the variability of the biliary system from the bile canaliculi to large bile ducts. The saliant findings of the present study include preserved localization of ECM proteins in the scaffold, efficient distribution of cells in the scaffold by slow and moderate speeds of perfusion culture, and bipotentiality of hepatocyte progenitors.

Hepatocytes and cholangiocytes differentiate from hepatocyte progenitors. During ductular reaction, progenitor hepatocytes around the Glissonean sheath in the injured liver reorganize the bile duct structure with help of factors such as tumor necrosis factor-α (TNF-α) and osteopontin [25, 26] *In vitro* experiments have confirmed that hepatocytes cultured in 3D collagen gel matrix form bile duct structures [27]. This phenomenon, similar to bile duct formation, can only be induced in hepatocytes under specific culture conditions and in presence of factors such as TNFα. To the best of our knowledge, none of the methods have succeeded in achieving effective delivery of stimulating factors to a cluster of hepatocyte organoids and facilitate their integration into a single dendritic structure. In this study, we assessed the utility of hepatocyte progenitors as alternative cell sources and the possibility of functional maturation of decellularized scaffolds. First, we investigated the localization of ECM proteins preserved in the liver scaffold using protein analysis, which revealed that the Glissonean area preserved different ECM proteins from those expressed in the liver parenchymal areas. The localization of ECM proteins was determined by their distribution in the native liver, indicating that the extracellular environment which is essential for the proper function of native livers, is preserved after decellularization.

We then optimized the methods for recellularizing ductal structures originating from bile ducts using primary cholangiocytes. Given that cholangiocytes differentiate from hepatocyte progenitors, we first used primary cholangiocytes to optimize an efficient protocol for repopulating cholangiocytes in decellularized scaffolds because they have similar cellular characteristics. We observed ductal, continuous arraying of CK19-positive primary cholangiocytes without discontinuation or congestion, which is essential for the normal function of the biliary system as a secretion route for bile acids.

Over the last decade, considerable research has been conducted on cell sources for organ reconstruction using decellularized scaffolds [24, 28, 29]. Most recently, Takeishi et al. reported that hepatocytes, cholangiocytes, and vascular endothelial cells established from human iPS cells recellularize into rat liver decellularized scaffolds that can be transplanted into rats, where they produce bile acids [22]. However, maintenance of iPS cells is challenging and costly. Additionally, iPS cell-derived mature cells cannot be used to recapitulate the local heterogeneity of the bile ducts, mainly because extrahepatic large bile ducts and small intrahepatic bile ducts arise separately and connect throughout the developmental stage [5]. Cholangiocytes lining the bile ducts are also express different transporters depending on their location, which makes bile duct regeneration more complicated [30]. Specifically, receptors such as SCTR, CAR2, and AQP-8 are expressed in large bile ducts, whereas receptors such as BSEP and CFTR are expressed in small bile ducts such as bile canaliculi [30–36]. To this end, reconstruction of the bile excretory pathway requires not only a structural increase in size from intrahepatic to extrahepatic, but also polarization of hepatocytes and each type of biliary epithelium. In this respect, hepatocyte progenitors with the potential to differentiate into both hepatocytes and cholangiocytes have the advantage of being a single resource for the reconstruction of complicated structures with both cell types.

Decellularized scaffolds have the size and stiffness that allow their transplantation into organs, and recellularization has succeeded in partially reproducing organ functions *in vitro* and *in vivo*. The original structure of blood vessels and bile ducts is maintained in the decellularized scaffold of the liver [21, 37, 38], and the ability to reproduce cellular localization similar to that of the native liver by recellularizing each cell from different perfusion methods has greatly advanced reproducing functional structures with multiple cells on an organ scale. Hassanein et al. showed that the bile duct can be used as a route for recellularization at a continuous perfusion speed of 25 mL/min and this approach achieves a uniform parenchymal distribution throughout the scaffold [39]. From the viewpoint of efficient repopulation of ductal structures originating from bile ducts and parenchymal areas near the bile ducts, the speed of perfusion culture should be slower to prevent hepatocyte progenitors from getting scattered in the peripheral parenchymal areas. We optimized the perfusion culture method with a speed of 300 μL/min for repopulating hepatocyte progenitors in parenchymal areas and 10 μL/min consecutive culture for the repopulation of ductal structures. This method successfully allowed the repopulation of cholangiocytes through the entire ductal structure. Moreover, progenitor hepatocytes differentiated into hepatocytes in the parenchymal area and into cholangiocytes in the ductal structure. This result indicates that ECM proteins locally preserved in each area stimulate hepatocyte progenitors and enhance cell differentiation. The preservation of ECM proteins in the scaffold, which enables progenitors to differentiate into mature cells depending on the location, is advantageous in organ-size regeneration techniques because it is difficult to deliver essential stimulants for the differentiation of progenitors and regeneration of organ structures to the appropriate regions in the decellularized scaffold. Furthermore, the expression of genes specific to multiple bile duct types (e.g. *BSEP* and *AQP8*), as well as hepatocytes, was increased in the recellularized scaffold with hepatocyte progenitors. *BSEP* is expressed in hepatocytes that form bile canaliculi, which are essential for the secretion of bile acid, whereas *AQP8* is a major gene in the intrahepatic bile duct. The gene expression profile obtained in our study suggests that the combining hepatocyte progenitors and decellularized scaffolds is a promising technique for functional reconstruction of the entire biliary system. Moreover, hepatocyte progenitors are relatively easy to handle compared to other cell sources, such as iPS cells, because they require less complicated and prolonged procedures to obtain sufficient number of cells for recellularization. In this study, the recellularization protocol was optimized to achieve reconstruction of the bile canaliculi and the junction between hepatocytes and cholangiocytes, rather than the uniform recellularization of the entire scaffold. Accordingly, the cells were distributed in the bile ducts and near the bile duct terminus, while the distribution of cells into the parenchymal area was sparse, resulting in large errors between samples. Notably, sophisticated techniques are needed to maintain a constant perfusion pressure and a homogeneous culture environment while preventing contamination during culture of the scaffolds with recellularised hepatocyte progenitors. Furthermore, the scaffold must be anatomically contiguous with the bile duct into which the catheter is inserted to distribute cells. Hence, while the establishment and culture management of hepatocyte progenitors is easier than that of iPS cells, the establishment and culture management of a large number of hepatocyte progenitors is somewhat challenging. In the present study, the size of the scaffold was reduced by shaping rat livers while maintaining the continuity of the bile ducts, however, a large number of hepatocyte progenitors would be required to seed cells throughout the scaffold. The inability to increase the number of samples and the variability of the results obtained posed difficulty in statistical analysis, and were considered the limitations of this method.

Other types of cells that do not originate from hepatocyte progenitors, such as vascular endothelial cells or Kupffer cells, are required to regenerate a fully functional liver. During this study, we attempted simultaneous perfusion culture of several cells, but it resulted in congestion of the injected cells in the bile duct. This is partially due to the difference in perfusion pressure in the bile duct and other vessels. Therefore, an optimal protocol is needed for the simultaneous recellularization of multiple cell types. From the viewpoint of clinical applications, Katsuda et al. recently successfully established human hepatocyte progenitors from human infant hepatocytes [40]. In addition, pre-clinical transplant experiments using existing large animal models of cirrhosis are necessary for real-world clinical implementation [41], hence our study demonstrated the potential of decellularized scaffolds with CLiPs as grafts for transplantation.

In conclusion, this study demonstrates an optimized method of hepatocyte progenitor recellularization in decellularized liver scaffolds via biliary ductal structure. Repopulation and redifferentiation of hepatocyte progenitors into hepatocytes and cholangiocytes were achieved, and the liver parenchyma and biliary system were morphologically reconstructed. In addition, the reconstructed biliary system expressed receptor genes unique to both bile canaliculi and large bile ducts, suggesting that the localization of ECMs in the scaffolds induced cell maturation suited for each site of the biliary ductal structure via inducing ECM-cell interactions. These findings have the implications for producing bioengineered organ grafts using decellularized organ scaffolds and organ progenitors.

## Supporting information

S1 Data (XLSX)

## References

[1] Ghonem NS, Assis DN, Boyer JL. Fibrates and cholestasis. Hepatology. 2015;62:635–643. doi: 10.1002/hep.27744 25678132 PMC4515188

[2] Shen WJ, Chen G, Wang M, Zheng S. Liver fibrosis in biliary atresia. World J Pediatr. 2019;15:117–123. doi: 10.1007/s12519-018-0203-1 30465125

[3] Jacquemin E. Progressive familial intrahepatic cholestasis. Clin Res Hepatol Gastroenterol. 2012;36:S26–S35. doi: 10.1016/S2210-7401(12)70018-9 23141890

[4] Tanimizu N, Mitaka T. Morphogenesis of liver epithelial cells. Hepatol Res. 2016;46:964–976. doi: 10.1111/hepr.12654 26785307

[5] Tanimizu N, Kaneko K, Itoh T, Ichinohe N, Ishii M, Mizuguchi T, et al. Intrahepatic bile ducts are developed through formation of homogeneous continuous luminal network and its dynamic rearrangement in mice. Hepatology. 2016;64:175–188. doi: 10.1002/hep.28521 26926046

[6] Hashimoto W, Sudo R, Fukasawa K, Ikeda M, Mitaka T, Tanishita K. Ductular network formation by rat biliary epithelial cells in the dynamical culture with collagen gel and dimethylsulfoxide stimulation. Am J Pathol. 2008;173:494–506. doi: 10.2353/ajpath.2008.071024 18583317 PMC2475786

[7] Korelova K, Jirouskova M, Sarnova L, Gregor M. Isolation and 3D collagen sandwich culture of primary mouse hepatocytes to study the role of cytoskeleton in bile canalicular formation in vitro. J Vis Exp. 2019;154. doi: 10.3791/60507 31904017

[8] Du Y, Polacheck WJ, Wells RG. Bile duct-on-a-chip. Methods Mol Biol. 2022;2373:57–68. doi: 10.1007/978-1-0716-1693-2_4 34520006 PMC9056007

[9] Koike H, Iwasawa K, Ouchi R, Maezawa M, Giesbrecht K, Saiki N, et al. Modelling human hepato-biliary-pancreatic organogenesis from the foregut-midgut boundary. Nature. 2019;574:112–116. doi: 10.1038/s41586-019-1598-0 31554966 PMC7643931

[10] Higashi H, Yagi H, Kuroda K, Tajima K, Kojima H, Nishi K, et al. Transplantation of bioengineered liver capable of extended function in a preclinical liver failure model. Am J Transplant. 2022;22:731–744. doi: 10.1111/ajt.16928 34932270 PMC9008767

[11] Niklason LE. Understanding the extracellular matrix to enhance stem cell-based tissue regeneration. Cell Stem Cell. 2018;22:302–305. doi: 10.1016/j.stem.2018.02.001 29499149 PMC5937845

[12] Zhu C, Coombe DR, Zheng MH, Yeoh GC, Li L. Liver progenitor cell interactions with the extracellular matrix. J Tissue Eng Regen Med. 2013;7:757–766. doi: 10.1002/term.1470 22467423

[13] Willemse J, van der Laan LJW, de Jonge J, Verstegen MMA. Design by nature: Emerging applications of native liver extracellular matrix for cholangiocyte organoid-based regenerative medicine. Bioengineering (Basel). 2022;9:110. doi: 10.3390/bioengineering9030110 35324799 PMC8945468

[14] Katoonizadeh A, Poustchi H. Adult hepatic progenitor cell niche: how it affects the progenitor cell fate. Middle East J Dig Dis. 2014;6:57–64. 24872864 PMC4034666

[15] Kojima H, Yagi H, Kushige H, Toda Y, Takayama K, Masuda S, et al. Decellularized organ-derived scaffold is a promising carrier for human induced pluripotent stem cells-derived hepatocytes. Cells. 2022;11:1258. doi: 10.3390/cells11081258 35455938 PMC9025569

[16] Farber E. Similarities in the sequence of early histological changes induced in the liver of the rat by ethionine, 2-acetylamino-fluorene, and 3’-methyl-4-dimethylaminoazobenzene. Cancer Res. 1956;16:142–148. 13293655

[17] Parent R, Marion MJ, Furio L, Trépo C, Petit MA. Origin and characterization of a human bipotent liver progenitor cell line. Gastroenterology. 2004;126:1147–1156. doi: 10.1053/j.gastro.2004.01.002 15057753

[18] Wright N, Samuelson L, Walkup MH, Chandrasekaran P, Gerber DA. Enrichment of a bipotent hepatic progenitor cell from naïve adult liver tissue. Biochem Biophys Res Commun. 2008;366:367–372. doi: 10.1016/j.bbrc.2007.11.129 18062915 PMC2277337

[19] Katsuda T, Kawamata M, Hagiwara K, Takahashi RU, Yamamoto Y, Camargo FD, et al. Conversion of terminally committed hepatocytes to culturable bipotent progenitor cells with regenerative capacity. Cell Stem Cell. 2017;20:41–55. doi: 10.1016/j.stem.2016.10.007 27840021

[20] Kim Y, Kang K, Lee SB, Seo D, Yoon S, Kim SJ, et al. Small molecule-mediated reprogramming of human hepatocytes into bipotent progenitor cells. J Hepatol. 2019;70:97–107. doi: 10.1016/j.jhep.2018.09.007 30240598

[21] Yagi H, Fukumitsu K, Fukuda K, Kitago M, Shinoda M, Obara H, et al. Human-scale whole-organ bioengineering for liver transplantation: A regenerative medicine approach. Cell Transplant. 2013;22:231–242. doi: 10.3727/096368912X654939 22943797 PMC3682787

[22] Takeishi K, Collin de l’Hortet A, Wang Y, Handa K, Guzman-Lepe J, Matsubara K, et al. Assembly and function of a bioengineered human liver for transplantation generated solely from induced pluripotent stem cells. Cell Rep. 2020;31:107711. doi: 10.1016/j.celrep.2020.107711 32492423 PMC7734598

[23] Kojima H, Yasuchika K, Fukumitsu K, Ishii T, Ogiso S, Miyauchi Y, et al. Establishment of practical recellularized liver graft for blood perfusion using primary rat hepatocytes and liver sinusoidal endothelial cells. Am J Transplant. 2018;18:1351–1359. doi: 10.1111/ajt.14666 29338127

[24] Li K, Tharwat M, Larson EL, Felgendreff P, Hosseiniasl SM, Rmilah AA, et al. Re-endothelialization of decellularized liver scaffolds: A step for bioengineered liver transplantation. Front Bioeng Biotechnol. 2022;10:833163. doi: 10.3389/fbioe.2022.833163 35360393 PMC8960611

[25] Fickert P, Thueringer A, Moustafa T, Silbert D, Gumhold J, Tsybrovskyy O, et al. The role of osteopontin and tumor necrosis factor alpha receptor-1 in xenobiotic-induced cholangitis and biliary fibrosis in mice. Lab Invest. 2010;90:844–852. doi: 10.1038/labinvest.2010.61 20368698 PMC4285781

[26] Wang X, Lopategi A, Ge X, Lu Y, Kitamura N, Urtasun R, et al. Osteopontin induces ductular reaction contributing to liver fibrosis. Gut. 2014;63:1805–1818. doi: 10.1136/gutjnl-2013-306373 24496779

[27] Nishikawa Y, Sone M, Nagahama Y, Kumagai E, Doi Y, Omori Y, et al. Tumor necrosis factor-α promotes bile ductular transdifferentiation of mature rat hepatocytes in vitro. J Cell Biochem. 2013;114:831–843. doi: 10.1002/jcb.24424 23097189

[28] Asadi M, Khalili M, Lotfi H, Vaghefi Moghaddam S, Zarghami N, André H, et al. Liver bioengineering: Recent trends/advances in decellularization and cell sheet technologies towards translation into the clinic. Life Sci. 2021;276:119373. doi: 10.1016/j.lfs.2021.119373 33744324

[29] Hillebrandt KH, Everwien H, Haep N, Keshi E, Pratschke J, Sauer IM. Strategies based on organ decellularization and recellularization. Transpl Int. 2019;32:571–585. doi: 10.1111/tri.13462 31099920

[30] Ueno Y, Alpini G, Yahagi K, Kanno N, Moritoki Y, Fukushima K, et al. Evaluation of differential gene expression by microarray analysis in small and large cholangiocytes isolated from normal mice. Liver Int. 2003;23:449–459. doi: 10.1111/j.1478-3231.2003.00876.x 14986819

[31] Keppler D, Konig J. Hepatic canalicular membrane 5: Expression and localization of the conjugate export pump encoded by the MRP2 (cMRP/cMOAT) gene in liver. FASEB J. 1997;11:509–516. doi: 10.1096/fasebj.11.7.9212074 9212074

[32] Pauli-Magnus C, Meier PJ. Hepatocellular transporters and cholestasis. J Clin Gastroenterol. 2005;39:S103–S110. doi: 10.1097/01.mcg.0000155550.29643.7b 15758645

[33] Koyama Y, Yamamoto T, Kondo D, Funaki H, Yaoita E, Kawasaki K, et al. Molecular cloning of a new aquaporin from rat pancreas and liver. J Biol Chem. 1997;272:30329–30333. doi: 10.1074/jbc.272.48.30329 9374520

[34] Körner M, Hayes GM, Rehmann R, Zimmermann A, Scholz A, Wiedenmann B, et al. Secretin receptors in the human liver: Expression in biliary tract and cholangiocarcinoma, but not in hepatocytes or hepatocellular carcinoma. J Hepatol. 2006;45:825–835. doi: 10.1016/j.jhep.2006.06.016 16935383

[35] Farouk M, Vigna SR, Haebig JE, Gettys TW, McVey DC, Chari R, et al. Secretin receptors in a new preparation of plasma membranes from intrahepatic biliary epithelium. J Surg Res. 1993;54:1–6. doi: 10.1006/jsre.1993.1001 8094102

[36] Alpini G, Ulrich C, Roberts S, Phillips JO, Ueno Y, Podila PV, et al. Molecular and functional heterogeneity of cholangiocytes from rat liver after bile duct ligation. Am J Physiol. 1997;272:G289–G297. doi: 10.1152/ajpgi.1997.272.2.G289 9124353

[37] Soto-Gutierrez A, Zhang L, Medberry C, Fukumitsu K, Faulk D, Jiang H, et al. A whole-organ regenerative medicine approach for liver replacement. Tissue Eng Part C Methods. 2011;17:677–686. doi: 10.1089/ten.TEC.2010.0698 21375407 PMC3103054

[38] Uygun BE, Soto-Gutierrez A, Yagi H, Izamis ML, Guzzardi MA, Shulman C, et al. Organ reengineering through development of a transplantable recellularized liver graft using decellularized liver matrix. Nat Med. 2010;16:814–820. doi: 10.1038/nm.2170 20543851 PMC2930603

[39] Hassanein W, Uluer MC, Langford J, Woodall JD, Cimeno A, Dhru U, et al. Recellularization via the bile duct supports functional allogenic and xenogenic cell growth on a decellularized rat liver scaffold. Organogenesis. 2017;13:16–27. doi: 10.1080/15476278.2016.1276146 28029279 PMC5323036

[40] Katsuda T, Matsuzaki J, Yamaguchi T, Yamada Y, Prieto-Vila M, Hosaka K, et al. Generation of human hepatic progenitor cells with regenerative and metabolic capacities from primary hepatocytes. eLife. 2019;8:e47313. doi: 10.7554/eLife.47313 31393263 PMC6731094

[41] Nishi K, Yagi H, Ohtomo M, Nagata S, Udagawa D, Tsuchida T, et al. A thioacetamide-induced liver fibrosis model for pre-clinical studies in microminipig. Sci Rep. 2023;13:14996. doi: 10.1038/s41598-023-42144-8 37696857 PMC10495379

